# Magnetic resonance imaging of the supra-cervical fetal membrane detects an increased risk of prelabor rupture of membranes

**DOI:** 10.3389/fendo.2022.1001538

**Published:** 2022-09-29

**Authors:** Wenxu Qi, Peinan Zhao, Wei Wang, Zichao Wen, Zhexian Sun, Wenjie Wu, Pamela Karen Woodard, Qing Wang, Robert C. McKinstry, Yong Wang

**Affiliations:** ^1^ Department of Radiology, Shengjing Hospital of China Medical University, Shenyang, China; ^2^ The Departments of Obstetrics and Gynecology, School of Medicine, Washington University in St. Louis, St. Louis, MO, United States; ^3^ Mallinckrodt Institute of Radiology, School of Medicine, Washington University in St. Louis, St. Louis, MO, United States; ^4^ The Department of Biomedical Engineering, Washington University in St. Louis, St. Louis, MO, United States; ^5^ School of Medicine, Washington University in St. Louis, St. Louis, MO, United States; ^6^ The Department of Electrical & Systems Engineering, Washington University in St. Louis, St. Louis, MO, United States

**Keywords:** fetal membrane, preterm birth, prelabor rupture of membranes, preterm prelabor rupture of membranes, magnetic resonance imaging

## Abstract

**Objective:**

In 10% of term deliveries and 40% of preterm deliveries, the fetal membrane (FM) ruptures before labor. However, the ability to predict these cases of premature rupture of membranes (PROM) and preterm premature rupture of membranes (PPROM) is very limited. In this paper, our objective was to determine whether a prediction method based on T2 weighted magnetic resonance imaging (MRI) of the supra-cervical FM could predict PROM and PPROM.

**Methods:**

This prospective cohort study enrolled 77 women between the 28th and 37th weeks of gestation. Two indicators of fetal membrane defects, including prolapsed depth >5 mm and signal abnormalities, are investigated for our prediction. Fisher’s exact test was used to determine whether prolapsed depth >5 mm and/or signal abnormalities were associated with PROM and PPROM. The sensitivity, specificity, positive predictive value, negative predictive value, and accuracy were calculated for prolapsed depth >5 mm, signal abnormalities, and the combination of prolapsed depth >5 mm and signal abnormalities.

**Result:**

Among 12 women with PROM (5 preterm and 7 term, prior to labor onset), 9 had membrane prolapse >5 mm and 5 had FM signal abnormalities. Among 65 women with rupture of membranes at term, 2 had membrane prolapse >5 mm and 1 had signal abnormalities. By Fisher’s exact test both indicators, membrane prolapse >5 mm and signal abnormalities, were associated with PROM (P<0.001, P<0.001) and PPROM (P=0.001, P<0.001). Additionally, membrane prolapse >5 mm, signal abnormalities, and the combination of the two indicators all demonstrated high specificity for predicting PROM (96.9%, 98.5%, and 100%, respectively) and PPROM (90.3%, 97.2%, and 100%, respectively).

**Conclusion:**

MRI can distinguish the supra-cervical fetal membrane *in vivo* and may be able to identify women at high risk of PPROM.

## Introduction

During pregnancy, the developing fetus is surrounded by the amnion and chorion, collectively called the fetal membrane (FM). In a healthy pregnancy, the FM remains intact until delivery at term, when the rupture of membranes (ROM) occurs either naturally during labor or is induced by the physician. However, in 10% of term deliveries the FM ruptures before labor onset, which is defined as a prelabor rupture of membranes (PROM) (1). In 40% of preterm deliveries, the FM ruptures before labor onset; called preterm prelabor rupture of fetal membranes (PPROM) (1).

In all cases of ROM, the FM appears to rupture at a region of focal weakness (2). Quintero et al. confirmed endoscopically that this weak FM region is commonly located directly over the internal cervical os (3). Additionally, McLaren et al. showed that this region had a thicker connective tissue layer and thinner cytotrophoblast and decidual layers than FM regions near the placenta or midway between the placenta and cervix (4). Features that contribute to FM rupture include localized FM defects, decreased amniotic collagen content, uterine irritability, genital tract infection, membrane stretch (e.g., by twin pregnancy), programmed amniotic cell death, and general decreased FM tensile strength (5, 6).

Currently, we do not have reliable methods to accurately predict PROM or PPROM in the literature. Although FM defects have been reported in ultrasound images, most studies have focused on FM thickness (7–10). Moreover, the most recent review on this topic noted that the data was insufficient to determine the association between FM thicknesses measured by ultrasound and PPROM. It was suggested the use of newer technologies could lead to progress in this area (6). Here, we used magnetic resonance imaging (MRI) with T2 weighted imaging (T2WI) to examine the FM in 77 women between 28 and 37 weeks of pregnancy. In our cohort, FM defects ascertained on MRI could predict PROM or PPROM with high accuracy.

## Materials and methods

### Enrollment

This study was part of a prospective cohort study using MRI to investigate the risk of preterm birth. The prospective cohort study and this study were approved by the Washington University in St. Louis School of Medicine Institutional Review Board (protocols 202006005,201612140 and 201707152). Participants were included if they were age 18 years or older and had a healthy singleton pregnancy. Participants were excluded if they had a twin pregnancy, contraindications to MRI, BMI over 40, major fetal anomalies, placenta implantation, or cervical cerclage. Between April 2017 and Feb 2020, 82 pregnant women were enrolled in the original study in the Department of Obstetrics and Gynecology Outpatient Clinic at Washington University School of Medicine. Written informed consent was obtained from each participant, including consent to future research. For the prospective cohort study, MRI images of the pelvis were collected at multiple time points during pregnancy. For this study, we used the last examination for each patient, performed between the 28^th^ and 37^th^ weeks of gestation.

### MRI Protocol

MR images were acquired with a 3.0 Tesla unit (Magnetom Vida; Siemens Medical Solutions, Erlangen, Germany). Patients were imaged in the left lateral position with their feet entering the magnet bore first. A 30-channel phased-array torso coil covered the entire pelvis. Each subject underwent MRI sequences that included axial and oblique sagittal planes T2 weighted (T2W) half-Fourier acquisition single-shot turbo spin-echo sequence images. The scanning parameters were as follows: repetition time 1800 ms, echo time 94 ms, matrix 320×650, layer thickness 4.0 mm, slice spacing 0.8 mm, number of layers 30, flip angle 140°. The axial and oblique sagittal T2W scanning fields of view were 400×400 mm and 350×350 mm, respectively. The total scanning time for T2WI was 3 minutes.

### Image analysis

**Figure 1:**
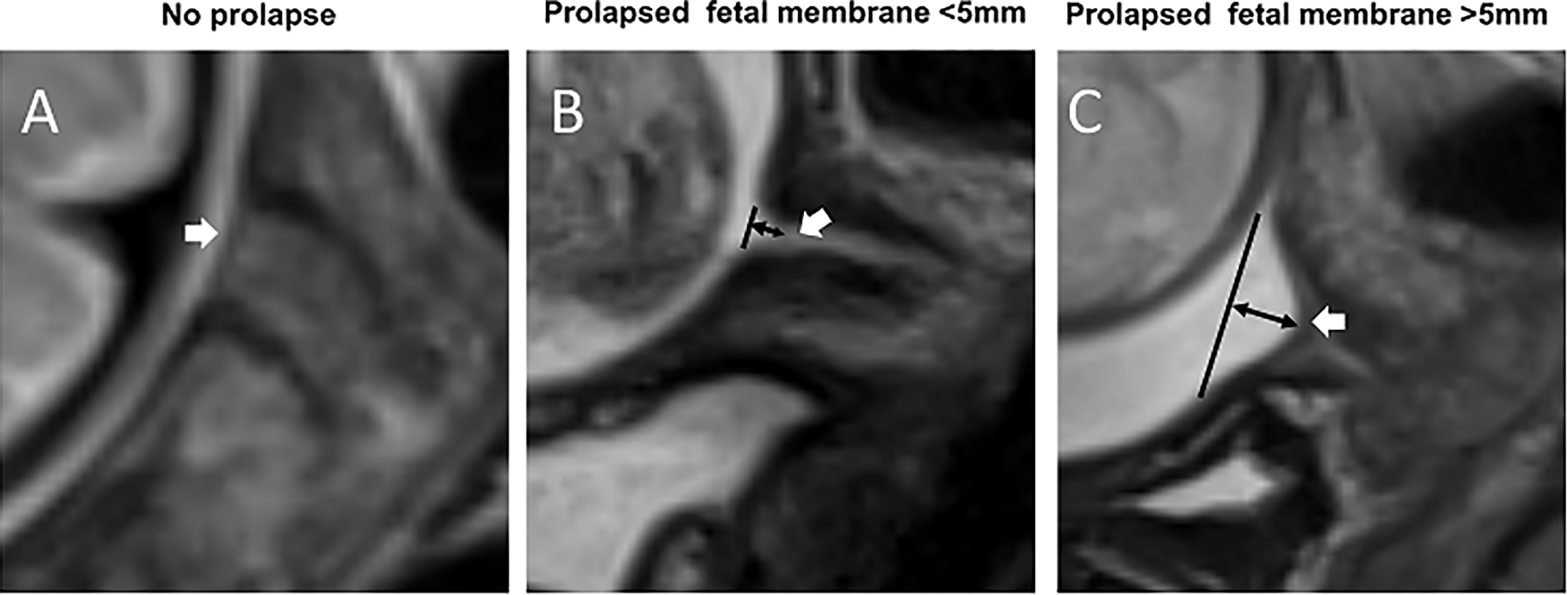
Prolapsed fetal membrane. T2WI of the cervix in the sagittal view. **(A)** A fetal membrane with no prolapse at 32 weeks’ gestation. **(B)** A prolapsed fetal membrane <5 mm at 32 weeks. **(C)** A prolapsed fetal membrane >5mm at 28 weeks. White arrows indicate the fetal membrane; black lines indicate the position of the internal cervical os, and black double arrows indicate the extent of membrane prolapse.

**Figure 2:**
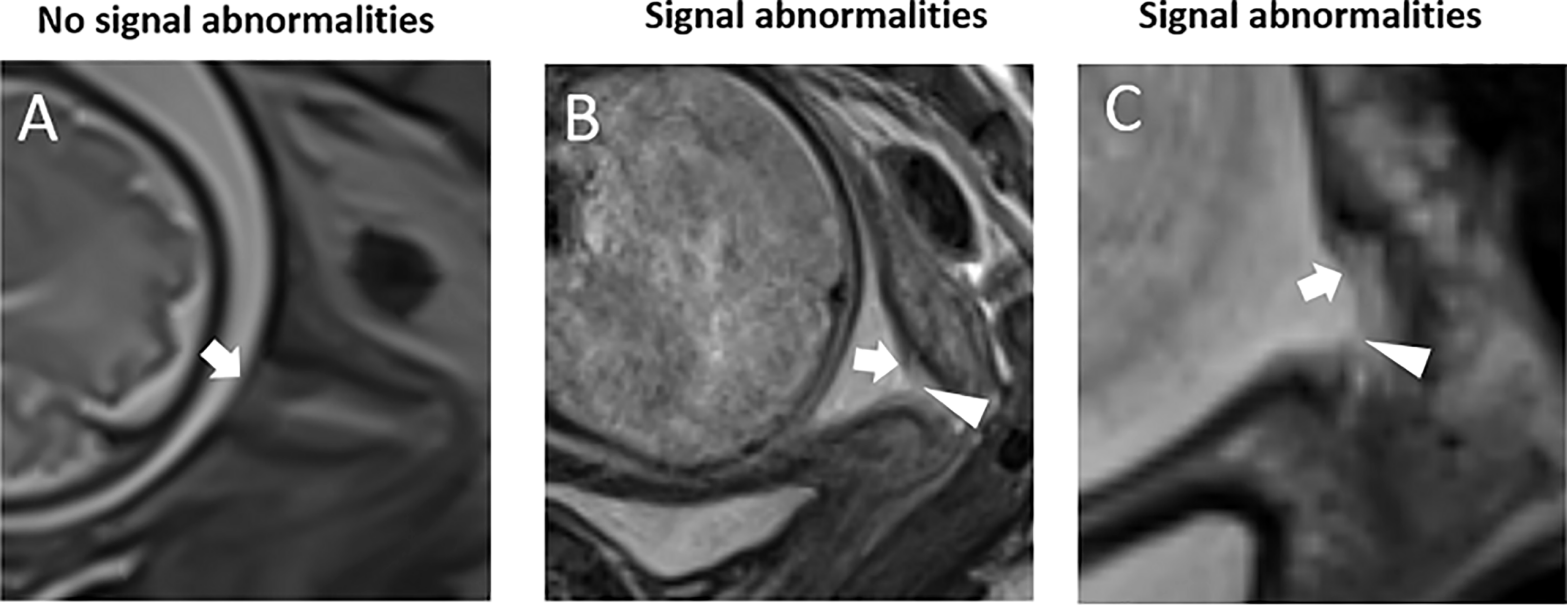
Fetal membrane signal abnormalities. T2WI of the cervix in the sagittal view. **(A)** A fetal membrane with no signal abnormalities at 32 weeks’ gestation. **(B)** Prolapsed fetal membrane with a signal abnormality – partial defect of the continuous fetal membranes signal – at 32 weeks. **(C)** Prolapsed fetal membrane with a signal abnormality – local thinning and increased signal intensity of the continuous fetal membrane signal – at 28 weeks. White arrows indicate the fetal membrane, and white triangles indicate fetal membrane defects.

RadiAnt DICOM viewer (version 4.6.8. for Windows, Medixant, Poland) was used to assess MR images of the cervix and FM. The images were analyzed without knowledge of pregnancy outcomes by two radiologists (W. Q. and W. W.) with 10-year and 1-year of experience in abdominal MRI, respectively. In order to examine the observer variability, the images were independently and blindly analyzed by the two observers at two different times with a minimum 60-day interval. The consensus was reached through in-person discussion and review in cases of disagreement. The following parameters of the FM overlying the cervix were evaluated: a prolapsed depth >5 mm, defined as a protrusion of amniotic membranes into the internal cervical os by greater than 5 mm from the shoulder of the original internal os as measured along the lateral border of the funnel (**Figure 1**)  (11); the presence of signal abnormalities, such as partial defect, local thinning, or local signal intensity increase (**Figure 2**).

### Demographic information

Clinical data recorded included the following: age, parity, last menstrual period dates, medical disorders, and past obstetric outcomes. Current pregnancy information collected included whether or not cerclage was placed, whether or not the woman was treated with progesterone, and any antepartum complications. Hospital records were reviewed after delivery to determine pregnancy outcome. The principal outcome was PROM, which included any rupture before labor (1). PPROM is defined as the FM ruptures before labor onset and before 37 weeks of gestation (1).

### Statistical analysis

Statistical analysis was performed with SPSS^®^ v. 19.0 for Windows^®^ (IBM Corp., New York, NY; formerly SPSS Inc., Chicago, IL) and GraphPad Prism (version 4.02 for Windows, GraphPad Software, San Diego, CA). Intra- and inter-observer agreements were calculated by means of the kappa index (κ). Reliability is rated as ‘moderate’ for values between 0.41–0.60, as ‘substantial’ for values between 0.61–0.8 and as ‘excellent’ for values above 0.80 (12). Fisher’s exact test was used to determine whether FM prolapse >5 mm and signal abnormalities are associated with PROM and PPROM. The sensitivity, specificity, positive predictive value, negative predictive value, and accuracy were calculated for those two indicators and the combination of FM prolapse and signal abnormalities. The statistical significance was set at *P* < 0.05.

## Results

Of the 82 patients for whom we had MR images from the retrospective study, one patient was excluded because she had hematocolpometra, which prevented observation of the cervical zone FM in MR images. Four patients were excluded because of preterm labor with intact membranes. The Demographic details of the 77 women included in this study are presented in **Table 1**.

**Table 1:** Demographics of the study population.

	Total (n = 77)	ROM at labor (n = 65)	PROM (n = 7)	PPROM (n = 5)
Age, years, median (range)	27 (18-37)	26.5 (18-37)	27 (19-31)	27 (25-28)
Body mass index, kg/m2, average (range)	26.5 (18.1-39.2)	26.6 (18.1-39.3)	24 (18.5-33.7)	29.3 (22.4-35.6)
Race/ethnicity, n (%)				
African American	48 (62.3)	42 (64.6)	3 (42.9)	4 (80)
Caucasian	26 (33.8)	23 (35.4)	2 (28.6)	1 (20)
Asian	1 (1.3)	0	1 (14.3)	0
Other	2 (2.6)	0	1(14.3)	0
Multiparous, n (%)	60 (77.9)	52 (80)	4 (57.1)	5 (100)
Previous preterm birth, n (%)	17 (22.1)	12 (18.5)	2 (28.6)	4 (80)
GA at MRI examination, median (range)	36 (22-37)	36 (32-37)	36 (32-37)	32 (22-32)
GA at deliver, median (range)	38 (28-40)	39 (37-40)	39 (37-40)	29.5 (28-36)
MRI examination to delivery interval,median (range)	3.5 (1-9)	4 (1-8)	4 (1-6)	4 (1-9)
Cervical length,cm,median (range)	3.1 (1.5-5.2)	3.2 (1.7-5.2)	2.95 (2.1-3.2)	2.15 (1.5-2.3)

The pregnancy outcomes were as follow: 12 women had PROM (15.6%, including 5 PPROM and 7 ROM at term prior to labor onset), and 65 women had term ROM (84.4%, including 32 spontaneous and 33 artificial).

T2W MRI datasets were collected from each of the 77 pregnant women. There were excellent or substantial Intra- and inter-observer agreement for the diagnosis of a prolapsed membrane >5 mm and signal abnormalities (**Table 2**). In the 12 women with PROM, nine (75%) had a prolapsed membrane >5 mm (**Figure 1**), five (41.7%) had FM signal abnormalities (**Figure 2**), and five women presented with both criteria. In the five women with PPROM, four (80%) had prolapsed membrane >5 mm and four (80%) had FM signal abnormalities. Three women had both criteria present. In the 65 women with term ROM, two (3.1%) had a prolapsed membrane >5 mm and one (1.5%) had FM signal abnormalities.

**Table 2:** Inter- and intra-observers agreement by Kappa statistics.

	Observer A		Observer B		First reading		Second reading	
	First reading and second reading		First reading and second reading		Observer A and Observer B		Observer A and Observer B	
	K	95% CI	K	95% CI	K	95% CI	K	95% CI
prolapsed membrane >5 mm	0.95	0.84-1.05	1		0.95	0.85-1.05	1	
signal abnormalities	0.78	0.54-1.02	0.80	0.62-0.99	0.72	0.47-0.98	0.75	0.54-0.96

CI, confidence interval.

By Fisher’s exact test, we conclude that a prolapsed membrane >5 mm and signal abnormalities are both associated with PROM (P<0.001, P<0.001) and PPROM (P=0.001, P<0.001). The abilities of these measures to predict PROM and PPROM are presented in **Tables 3**, **4**. All FM indicators presented high specificity.

**Table 3:** Ability of MRI-detected FM defects to predict PROM*.

	Sensitivity, % (n)	Specificity, % (n)	Positive predictive value, % (n)	Negative predictive value, % (n)	Accuracy, % (n)
Prolapsed membrane >5mm	75 (9/12)	96.9 (63/65)	81.8 (9/11)	95.5 (63/66)	93.5 (72/77)
Signal abnormalities	41.7 (5/12)	98.5 (64/65)	83.3 (5/6)	90.1 (64/71)	89.6 (69/77)
Combination of prolapsed membrane >5mm and signal abnormalities	50 (6/12)	100 (65/65)	100 (4/4)	89.0 (65/73)	89.6 (69/77)

*For this analysis, PROM was defined as any rupture of membrane before labor.

**Table 4:** Ability of MRI-detected FM defects to predict PPROM.

	Sensitivity, % (n)	Specificity, % (n)	Positive predictive value, % (n)	Negative predictive value, % (n)	Accuracy, % (n)
Prolapsed membrane >5mm	80 (4/5)	90.3 (65/72)	36.4 (4/11)	98.5 (65/66)	90.0 (69/77)
Signal abnormalities	80 (4/5)	97.2 (70/72)	66.7 (4/6)	98.6 (70/71)	96.1 (74/77)
Combination of prolapsed membrane >5mm and signal abnormalities	60 (3/5)	100 (72/72)	100 (3/3)	97.3 (72/74)	97.4 (75/77)

## Discussion

This study provides evidence that two FM indicators derived from MRI can be used to predict PROM and PPROM. This is the first study to demonstrate the feasibility of using MRI to assess supra-cervical FM. These two MRI features, membrane prolapse >5 mm and FM signal abnormalities, reflect the membrane stretch under unbalanced load and/or pre-weakened FM, and likely indicate the increased risk of PROM and PPROM.

First, we examined prolapse. In a healthy pregnancy, the FM lies above the internal cervical os without prolapse because the mechanical pressures above and below the FM are sufficiently balanced. However, the FM will stretch under an unbalanced load (5, 13). If the bottom to top pressure decreases (e.g. cervical dilation, cervical insufficiency, or weak FM tension), or the top to bottom pressure increases (e.g. twin pregnancies or distended uterine volume), the FM will prolapse into the internal cervical os. A study comparing the FM surface area *in vivo* before delivery to the FM surface area *ex vivo* after delivery revealed that the FM is stretched *in vivo* by approximately three-fold and is under approximately 15 mmHg pressure (14). Furthermore, this study showed that the FM would rupture at approximately 34 mmHg, corresponding to an areal stretch of 4.681 (14). It has been reported that ultrasound-detected FM prolapse greater than 5 mm can be used as an indicator to diagnose cervical insufficiency in the second trimester (11, 15, 16). Our data is consistent with these reports. However, ultrasound can only evaluate the collapse of the fetal membrane, but no other abnormalities of the collapsed membrane, such as local thinning or partial defect. Our MRI results show that the membrane morphology and signal intensity also change dramatically among patients with cervical funneling, and can serve as important MRI features to improve the evaluation of the supra-cervical FM.

The second FM indicator we examined was FM signal abnormalities, which reflect FM structural defects. Such defects ultimately result in PROM (5, 13), as the stretch force generated by uterine contractions alone cannot rupture a normal FM (14). In sagittal MR images, a normal FM appears as a continuous linear structure that separates the amniotic fluid from the cervix. In a normal FM, collagen and connective tissue contribute to the continuous linear low/intermediate T2WI signal. However, an FM structural defect is visible in T2WI as discontinuous signals of the linear structure between the amniotic fluid and the cervix. A focal FM defect may be caused by disruption of the FM connective tissue, and an increased FM signal intensity may be caused by swelling of connective tissue. Previous *in vitro* studies of FM demonstrated that the rupture site is more likely than other regions to show altered morphology characterized by swelling, disruption of the connective tissue, and thinning of the trophoblast layer (2, 4, 17–23).

**Figure 3:**
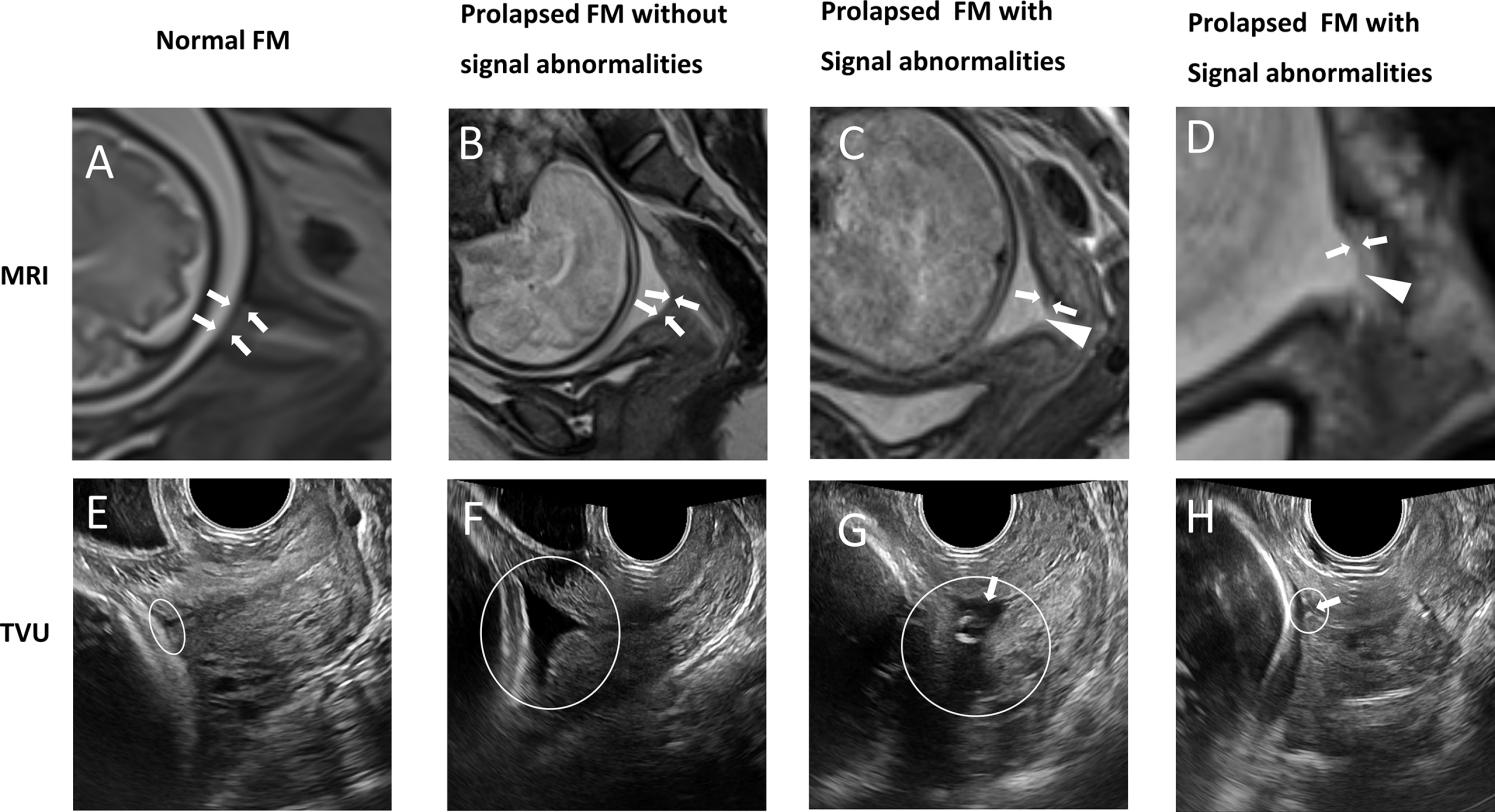
Patients underwent clinical MRI and transvaginal ultrasound (TVU) examinations on the same day. A vs. E (32wks); B vs. F (32wks); C vs. G (32wks); D vs. H (28wks). Panel **(A–D)** are MRI T2W images from the sagittal view. White arrows indicate the supra-cervical FM, and white triangles indicate fetal membrane defects. **(A)** Normal FM. **(B)** Prolapsed FM without signal abnormalities. **(C)** Prolapsed FM with signal abnormalities: partial defect of continuous FM signal. **(D)** Prolapsed FM with signal abnormalities: local thinning and increased signal intensity of continuous FM signal. The FM overlying the cervix is pointed by a white arrowhead. Panel **(E–H)** are TVU images. White rings indicate cervical internal os. **(E)** In the patient without prolapsed FM, the entire supra-cervical FM cannot be distinguished clearly due to the compression effect of the ultrasound probe. **(F)** In the patient with the prolapsed FM, the exact boundary between the supra-cervical FM and the underlying cervical tissue cannot be identified. **(G, H)** In patients with the prolapsed fetal membrane, the supra-cervical FM is partially visible. However, the signal abnormalities of the supra-cervical FM cannot be detected. White arrows point to the FM.

Previous studies have shown that the supra-cervical FM can occasionally be detected by ultrasound in the presence of chorioamniotic membrane separation (6, 24, 25). However, no detailed biological features of supra-cervical FM have been imaged and reported using ultrasound. It is technically challenging to identify FM near the internal os using transvaginal sonography (8). The reasons are two-fold. First, too much pressure from the ultrasound probe forces the anterior and posterior walls of the cervix to adhere together. The compressed internal os reduces the contact area between the FM and the cervical fluid below it. Second, ultrasound waves are reflected at the interface between media with different density (26). If there is no chorioamniotic membrane separation, we cannot observe the interface between the FM and the cervical tissue below it. In comparison, clinical MRI is able to reliably image the entire supra-cervical FM without deforming the cervical internal os. In addition, the supra-cervical FM is surrounded by the superior amniotic fluid, and inferior cervical fluid, both of which have high liquid T2WI signals, and thus can serve as natural contrast agents to highlight the FM (intermediate or low signal). When comparing MR images and transvaginal ultrasound images of the same patient on the same day (**Figure 3**), it is clear that MRI provides superior tissue contrast and biological characteristic information. In our previous study, we used 3D-CISS sequence to examine the fetal membrane at the cervical internal OS zone (27). Although 3D CISS sequence offers higher spatial resolution, it is not a routine clinical sequence, and not widely used in each of the clinical imaging session.

## Conclusion

Limitations of our study include the small sample size and potential selection bias (e.g. 22.1% of patients in our cohort had a history of preterm birth). Nonetheless, our study identified two potential MRI-based risk factors of FM rupture: pre-weakened FM, as reflected by signal abnormalities, and abnormal stretch force upon FM, as reflected by FM prolapse. This study suggests that clinical MRI can be used to identify women at increased risk of PPROM. Further studies with larger sample sizes are needed to evaluate the reproducibility of our findings and provide information for future comparative effectiveness and cost-effectiveness studies. Moreover, further MRI analyses may be able to identify women at high risk of PPROM, so as to provide appropriate antepartum surveillance and antenatal treatment.

## Data availability statement

The original contributions presented in the study are included in the article/supplementary material. Further inquiries can be directed to the corresponding author.

## Ethics statement 

The prospective cohort study and this study were approved by the Washington University in St. Louis School of Medicine Institutional Review Board (protocols 202006005,201612140 and 201707152). The patients/participants provided their written informed consent to participate in this study.

## Author contributions

WQ and PZ designed the experiment. WQ and WeiW evaluated magnetic resonance images. ZS, WenW, PW, and QW collected the data and aided in preparation of the manuscript. RM co-supervised the research. YW obtained funding for the project, supervised the work, and participated in preparation of the manuscript. All authors contributed to the article and approved the submitted version.

## Acknowledgments

We thank Deborah Frank for editing the manuscript. We also thank Jessica Chubiz and Megan Steiner for coordinating the clinical team to enroll patients.

## Conflict of interest

The authors declare that the research was conducted in the absence of any commercial or financial relationships that could be construed as a potential conflict of interest.

## Publisher’s note

All claims expressed in this article are solely those of the authors and do not necessarily represent those of their affiliated organizations, or those of the publisher, the editors and the reviewers. Any product that may be evaluated in this article, or claim that may be made by its manufacturer, is not guaranteed or endorsed by the publisher.
